# Phenotypic variability and the gender paradox in the R363C variant of Fabry disease

**DOI:** 10.1002/jmd2.12466

**Published:** 2025-01-16

**Authors:** Alison C. Leslie, Jeanine Jarnes, Alia Ahmed, Sofia Shrestha, Jeffrey Wang, Chester B. Whitley, Nishitha R. Pillai

**Affiliations:** ^1^ University of Minnesota Medical School Minneapolis Minnesota USA; ^2^ Division of General Internal Medicine Hospital of the University of Pennsylvania Philadelphia Pennsylvania USA; ^3^ Division of Genetics and Metabolism, Department of Pediatrics University of Minnesota Minneapolis Minnesota USA; ^4^ Advanced Therapies Clinic University of Minnesota Minneapolis Minnesota USA; ^5^ Division of Nephrology Hennepin County Medical Center Minneapolis Minnesota USA

**Keywords:** Fabry disease, genetic modifiers, kidney disease, Lyso‐GL‐3, phenotypic variability

## Abstract

Fabry disease is an X‐linked lysosomal disease caused by variants in the *GLA* gene. Although Fabry disease is X‐linked, *GLA* gene variants in females can exhibit a wide range of symptoms, challenging the traditional view of Fabry as an X‐linked recessive disease. A family is presented here with a 36‐year‐old female who is symptomatic with chronic kidney disease and her oligosymptomatic 70‐year‐old father, both of whom have a heterozygous and hemizygous GLA pathogenic variant, respectively, c.1087C>T (p.R363C). Interestingly, the proband's Lyso‐GL‐3 levels were lower than her father's despite her more severe clinical presentation. The discordance between clinical severity and Lyso‐GL‐3 levels, particularly in the context of migalastat therapy, raises questions about the appropriate interpretation and use of this biomarker. The earlier and more severe symptom onset in the female proband suggests the potential role of genetic modifiers or other factors influencing disease expression. This report underscores the complexity of Fabry disease phenotypes and the limitations of current biomarkers in predicting disease severity, particularly in females. The observed paradox between clinical symptoms and biomarker levels suggests the need for a deeper understanding of the underlying mechanisms driving phenotypic variability in Fabry disease.

**Synopsis**

There can be significant phenotypic variability in Fabry disease and this case report highlights the possibility of genetic modifiers leading to this phenotypic variability.

## 1 INTRODUCTION

Fabry disease is an X‐linked lysosomal disorder of the GLA gene on chromosome Xp22.1, encoding alpha‐galactosidase (alpha‐Gal A). Deficiency of this enzyme results in abnormal glycosphingolipid degradation in cell membranes, causing accumulation of globotriaosylceramide (Gb3/GL‐3) in vital organs and in tissues throughout the body.^1^ Female carriers of Fabry disease were initially presumed to be asymptomatic or to have a milder phenotype due to X‐inactivation. However, increasing evidence demonstrates that female carriers have a wide range of clinical symptoms, with some as severely affected as males.^2^ In a cohort of females with Fabry disease, nearly all (40/44) had evidence of Fabry disease affecting multiple organ systems. Renal manifestations were common including proteinuria in 56% and microalbuminuria in 80%.^3^ Female heterozygotes develop symptoms of Fabry disease at a much higher rate than could be explained by skewed X‐inactivation alone. It is therefore suggested that Fabry disease should no longer be classified as an “X‐linked recessive” disease, but rather as an “X‐linked” condition, and that female heterozygotes should not be termed “carriers” since that suggests a lack of symptoms and may delay evaluation of females with *GLA* variants.^3^

Because of the presence of private variants, genotype–phenotype correlation for many variants associated with Fabry disease is poor.^4^ Even within the same family, there is wide variability in target organ involvement and severity of disease.^5^ This suggests that the phenotypic variability with Fabry disease is likely mediated by other factors. Proposed mechanisms include polymorphisms in the promoter region of the *GLA* gene, the presence of modifier genes, epigenetic regulation of gene expression, and environmental factors, but these mechanisms have not been well studied.^5^ Fabry disease in a family is presented here, where the female proband has a much more severe clinical phenotype at a younger age than her oligosymptomatic father with the same variant. To our knowledge, this is the first report of a symptomatic female with the same genotype as an oligosymptomatic male within the same family and adds to the growing literature of great heterogeneity of clinical symptoms even with the same *GLA* variant.

## 2 PRESENTATION

### 2.1 Proband

Our proband is a now 36‐year‐old female with Fabry disease diagnosed at age 31 by Foresight Carrier Screening panel. The diagnosis was made after finding diminished ovarian reserve and selection of her sister as an egg donor. On carrier screening, her sister was found to have a *GLA* variant, and our proband was subsequently tested. Myriad Foresight Carrier Screen repeated for our proband was positive for a heterozygous *GLA* pathogenic variant: c.1087C>T; p.R363C, amenable to oral migalastat therapy. The sequencing and deletion/duplication analysis for other X‐linked genes showed no abnormalities, and her FMR trinucleotide repeat testing was also normal. Molecular testing did not detect any mosaicism.

**FIGURE 1:**
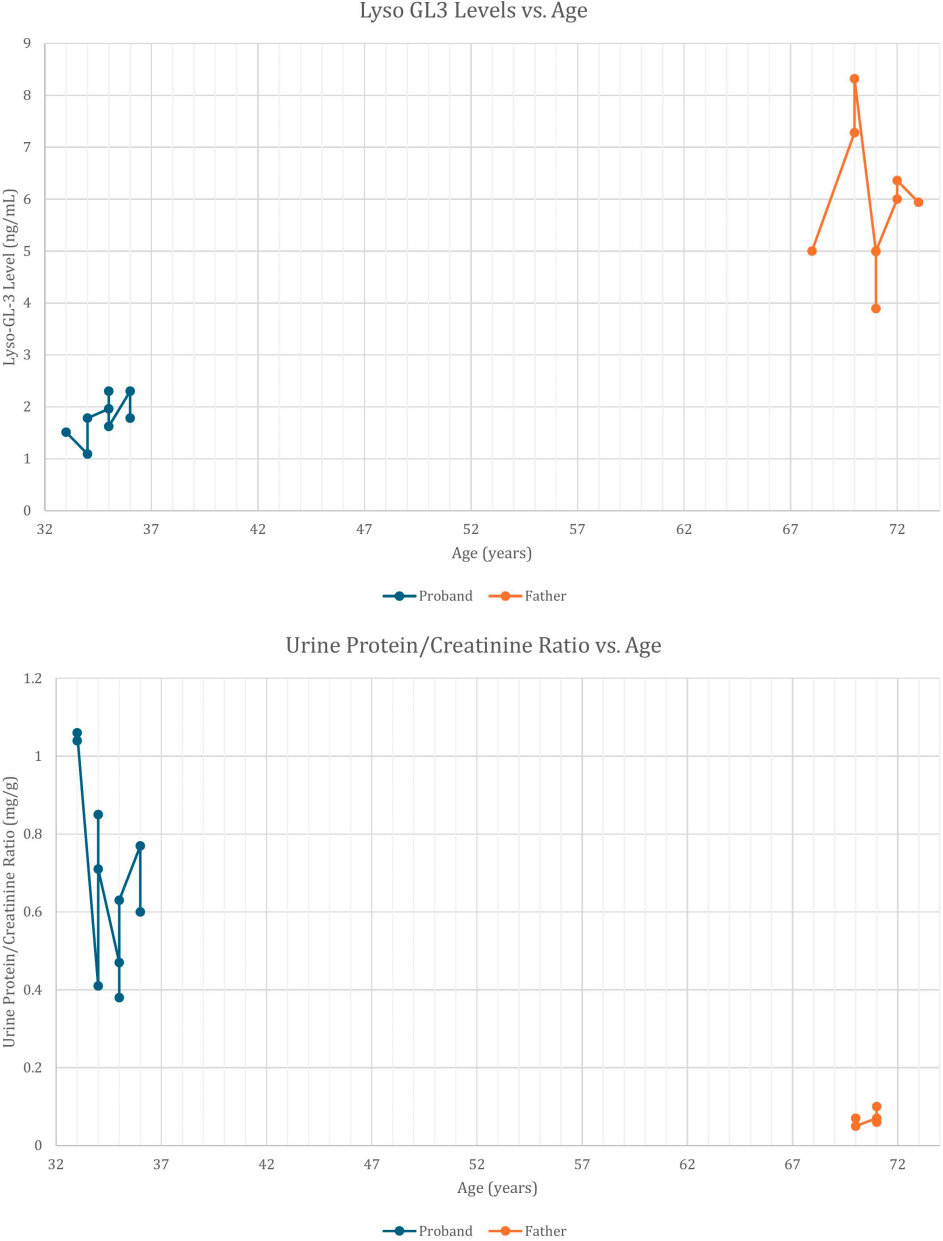
Lyso‐GL3 levels and urine protein/creatinine ratio plotted against age for the proband and her father. The proband (blue) and the father (orange) are represented on two separate graphs: One showing Lyso‐GL‐3 levels (top) and the other showing urine protein/creatinine ratio (bottom). The proband demonstrates lower Lyso‐GL‐3 levels compared with her father despite earlier and more severe clinical symptoms. The urine protein/creatinine ratio is elevated in the proband while remaining low in her oligosymptomatic father. This graph highlights the phenotypic variability between the two individuals, both carrying the same GLA variant.

She has a history of chronic kidney disease (CKD) type I due to persistent proteinuria with estimated glomerular filtration rate (eGFR) > 90 mL/min/1.73 m^2^ that was initially diagnosed at 31 years. There was no significant prior medical history indicative of renal injury. A renal biopsy 1 year later showed features consistent with Fabry nephropathy, including enlarged podocytes with foamy cytoplasm, ~30% podocyte foot process effacement, and the characteristic whorled zebra body inclusions in podocytes. Initial baseline creatinine at the time of diagnosis was 0.6–0.7 mg/dL, and increased to 1 mg/dL during pregnancy. At 36 years, her Lyso‐GL3 is 2.30 ng/mL with a baseline creatinine of 0.8–0.9 mg/dL and an eGFR of 90–100 mL/mg/1.73 m^2^ (Figure 1).

Cardiac MRI with late gadolinium enhancement at 33 years showed no evidence of infiltrative disease and there was no evidence of left ventricular (LV) hypertrophy on echocardiogram at 35 years. She does have mixed hyperlipidemia due to suspected familial hyperlipidemia, which was first diagnosed at age 32, and this condition is treated with rosuvastatin. At 34 years, she started having vision symptoms, including glare and halos, but at age 36, no cataracts or cornea verticillata were noted by ophthalmology.

She has been adherent on migalastat since beginning this therapy at 34 years of age and is tolerating the medication well. Physical examination was notable for abdominal angiokeratomas with 4–5 red spots on the abdomen, which her reports are similar to her father's report. She has no history of neurological symptoms, including headache or stroke. She has no history of chronic GI symptoms.

### 2.2 Father

The proband's father sought genetic testing following the positive diagnosis of Fabry disease in his daughter. His α‐Gal A level was mildly reduced at 0.032 U/L (ref 0.074–0.457), and subsequent genetic testing at 68 years confirmed the *GLA* variant of c.1087C>T; p.R363C without any evidence of mosaicism. He is completely asymptomatic except for angiokeratomas on his abdomen. Cardiac MRI was negative for Fabry involvement, and echocardiogram at 70 years showed normal LV function. He was diagnosed with cataracts at age 64 but no cornea verticillata. He has been on migalastat since 70 years of age. Despite a Lyso‐GL3 level being higher than his daughter's (Figure 1), he has no CKD, proteinuria, CVA, neuropathy, anhidrosis, or heart failure.

## 3 DISCUSSION

This report highlights familial Fabry disease associated with the GLA p.R363C variant (c.1087C>T), which leads to a late‐onset form of Fabry disease. Several publications identify this variant as associated with late‐onset disease; however, detailed phenotypic information is not available.^6^, ^7^, ^8^, ^9^ Mauhin et al. describe five individuals with this variant who exhibited no evidence of any end organ damage.^10^ A different variant affecting the same amino acid c.1088G>A; p.R363H has been associated with increased risk for a renal phenotype.^11^, ^12^, ^13^ Interestingly, the R363H variant has been associated with both the classic and the late onset form.^14^, ^15^, ^16^ The patient described here highlights that it is possible for females to experience more severe symptoms and earlier onset than males with the R363C variant.

Globotriaosylsphingosine (Lyso‐GL3) is a substrate of α‐Gal A that has emerged as a key biomarker for monitoring the progression of Fabry disease and the effects of enzyme replacement therapy (ERT), though it is not a good predictive biomarker for kidney involvement.^17^, ^18^ In females, diagnostic detection of Fabry disease is dependent on molecular diagnostics, elevated Lyso‐GL3, and Fabry symptomatology, and elevated Lyso‐GL3 is a stronger indicator of disease than low enzyme activity.^19^ Females with Fabry disease who started treatment based on clinical manifestations had higher Lyso‐GL3 and analogue biomarker levels than never‐treated females. This indicates that a biomarker cut‐off could potentially be a decision tool for treatment initiation in females with Fabry disease.^20^ However, in patients on migalastat, Lyso‐GL3 may not be an adequate biomarker for evaluating treatment response.^21^

ERT with agalsidase beta (Fabrazyme) improves renal function in males and females by reducing podocyte glycolipid deposits and decreasing circulating Lyso‐GL3 levels.^22^ Early ERT can delay progression to end‐stage renal disease in Fabry patients^19^ and reduce severe clinical events, including renal failure.^20^ Trends in eGFR suggest ERT maintains the normal age‐related decline in kidney function in females.^2^ Oral migalastat therapy offers a similar safety profile to ERT. In a single‐center study of 7 males, Lyso‐GL3 levels significantly decreased after 1 year of ERT but remained stable after switching to migalastat for 1 year, with proteinuria significantly lower during migalastat therapy.^21^ These findings suggest migalastat may offer important clinical benefits in Fabry disease especially for the renal phenotype.

Both our female proband and her father elected to be treated with migalastat chaperone therapy. In response to therapy, beginning at 34 years of age, the proband experienced a decrease in urine protein/creatinine ratio while the father's ratio remained unchanged and in the normal range. The proband has multi‐organ involvement, including CKD and proteinuria, beginning in her early 30s, while her father with the same variant (now in his 70s) has remained oligosymptomatic. This kind of phenotypic variability within the same family has not been previously described, leading to questions regarding whether gene modifiers are playing a role resulting in an earlier phenotype in our female proband. Additional testing, such as exome sequencing, will be pursued in this family, pending insurance approval.

Research studying the potential of genetic modifiers in Fabry disease is limited. Altarescu et al.^22^ have described the presence of various DNA polymorphisms in inflammatory and coagulation system‐related genes that appear to be associated with an increased risk of cerebral lesions and strokes in patients with Fabry disease. In other lysosomal diseases such as Niemann‐Pick disease type C, a polymorphism associated with decreased expression of sterol O acetyltransferase 1 (SOAT1) appears to be a genetic modifier of the type C1 phenotype and is associated with an earlier age of onset and higher frequency of liver disease and seizures. Further studies evaluating potential genetic modifiers of the Fabry disease phenotype are warranted.

The patients described herein are striking in that: (1) the female proband is symptomatic, and her father is oligosymptomatic; (2) The female proband showed lower disease biomarkers compared to her oligosymptomatic father. This paradox raises questions about the reliability of Lyso‐GL3 as a biomarker and the influence of genetic modifiers in Fabry disease. Additional research is needed to evaluate the phenotypic variability observed in Fabry disease.

## AUTHOR CONTRIBUTIONS

A. L. and N. R. P. involved in conceptualization, initial drafting and review of the manuscript. A. A., J. J., S. S., J. W., and C. W. edited and reviewed the manuscript.

## CONFLICT OF INTEREST STATEMENT

The authors declare no conflicts of interest.

## ETHICAL STATEMENT

IRB approval was not needed as this is a case report involving less than three patients.

## INFORMED CONSENT

Written informed consent was obtained from the patient for publication of this case report and any accompanying images.

## Data Availability

Due to the nature of the case report and to protect the privacy of the patient, some of the data are not publically available. Any inquiries regarding data sharing may be directed to the corresponding author, subject to institutional and ethical guidelines.
